# The role of learner character strengths and classroom emotions in L2 resilience

**DOI:** 10.3389/fpsyg.2022.956216

**Published:** 2022-10-14

**Authors:** Fakieh Alrabai, Abdullah Alamer

**Affiliations:** ^1^Department of English, Faculty of Languages and Translation, King Khalid University, Abha, Saudi Arabia; ^2^Department of English, King Faisal University, Alhasa, Saudi Arabia

**Keywords:** effort, emotional intelligence, negative emotions, positive psychology, positive emotions, resilience

## Abstract

This study aimed to examine a theory-driven model to explain how language learner's trait emotional intelligence (TEI) and effort as two learner character strengths predict learner enjoyment as a positive emotion and anxiety and boredom as two negative classroom emotions, and how these variables, collectively, predict resilience in language learning. The underlying relationship between these variables was tested *via* a comprehensive model within a positive psychology perspective using the partial least squares structural equation modeling (PLS-SEM) approach. The paths in the final structural model indicated that L2 learner TEI did not significantly explain their resilience directly but rather completely indirectly through the mediation of learner negative and positive emotions. Learner effort, directly and indirectly, predicted L2 resilience and its predictive power in it was much larger than that of TEI. In addition, enjoyment and boredom directly influenced L2 resilience and also mediated the relationship between learner character strengths and resilience. Anxiety did not significantly predict learner L2 resilience directly since its influence was rather dependent on the role of enjoyment and boredom in L2 resilience. These findings widely support the claims within positive psychology domain that recognize the vital role of character strengths and learner emotions in enhancing L2 learner resilience.

## Introduction

Based on the view of Seligman and Csikszentmihalyi (2014), the notion of positive psychology (PP) was founded on three pillars: (1) positive experiences/emotions, (2) positive personality character traits/strengths, and (3) positive institutions. Emotions; the first PP pillar; are multifaceted affective, physiological, behavioral, and cognitive reactions to the different situations experienced in learning situations (Bielak and Mystkowska-Wiertelak, 2020). According to the broaden-and-build theory (Fredrickson, 2001, 2009), emotions can be classified into two groups: positive (PEs) and negative (NEs). These different emotions serve different functions and usually have opposing impacts on learning. According to Fredrickson (2001, 2009), NEs such as anxiety and boredom restrict learner experience, decrease resilience and narrow down focus; whereas PEs like enjoyment broaden experience, build future emotional and cognitive resources (Bielak and Mystkowska-Wiertelak, 2020), and enhance learner resilience (Dewaele and MacIntyre, 2014).

Character strengths are the second pillar of positive psychology (Seligman and Csikszentmihalyi, 2014). The VIA inventory of character strengths (see Park et al., 2004) comprises a list of 24 character strengths classified under 6 broad virtues. Under the virtue of courage in this inventory is the character strength of persistence which involves, according to Kim and Kim (2017), maintaining effort by learners to solve problems in the face of obstacles and difficulties. In light of this definition, effort is a character strength that empowers learners to successfully acquire and learn the language (Hiver et al., 2021; Alamer, 2022a). In the same vein, emotional intelligence (EI), which is conceptualized by Salovey et al. (2009) as the ability to understand feelings in the self and others and to use these feelings as informational guides for thinking and action, is viewed as another key concept of character strengths within PP domain and so is its sub-component trait emotional intelligence (TEI) which is conceived by Petrides et al. (2007, p. 449) as “a constellation of emotional self-perceptions located at the lower levels of personality hierarchies.” Persistent effort in language learning is conceptualized by Lake (2013) as “the amount of time and frequency one spends studying the L2 and persisting in the face of obstacles and difficulties” (p. 230) and TEI have been found to be closely linked to learner emotions. However, the role of these two character strengths in language learner resilience, which is, according to Kim and Kim (2019, p. 3), “the ability to bounce back from adversity,” is yet to be established in second language research. So, relative to the volume of research defining the role of character strengths on LL and because the relationship between L2 learners' character strengths and classroom emotions identified by previous studies was established solely based on the findings of correlational studies, and also due to the lack of available research on the association between L2 learner character strengths and resilience, investigating the nature of this relationship in greater depth is crucial. This relationship requires to be investigated in one model that would allow unveiling the underlying processes in the relationship among learner character strengths, classroom emotions, and L2 resilience and therefore meaningfully substantiate the mechanisms that underlie such relationship. Hence, by using a structural equation modeling (SEM) approach we examine a theory-driven model to explain how two character strengths (TEI and effort) predict the positive and negative classroom emotions, and how these variables, collectively, predict resilience in language learning. This study has focused on efforts and TEI among the character strength because of the strong connections they have with learner emotions and L2 resilience as will be presented and discussed in the subsequent section of this article. In this regard, the findings of such an examination should support the theoretical claims that the concepts of TEI and effort are inherently emotion-related constructs and that learner emotions are usually subject to both learner-internal (e.g., TEI and effort) and learner-external regulators as suggested by Dewaele and MacIntyre (2019). A significant contribution of the present study is that it includes, besides learner emotions, learner character strengths and resilience as two important themes that have rarely been discussed in relation to language learning (Oxford, 2016). To the author's best knowledge, this study is among the first attempts in the L2 domain to investigate the interrelationship between TEI, learner emotions, and learner resilience in a single model. The study findings are anticipated to unveil the complex relationship between EFL learner character strengths and emotions and the role of these variables in predicting learner resilience for language learning.

## Literature review

### Trait emotional intelligence

TEI is one component of EI besides ability and emotional intelligence. Wellbeing, emotionality, self-control, and sociability are the four sub-factors of general TEI (see Petrides and Furnham, 2003). While wellbeing refers to being happy with life, demonstrating self-confidence, and being optimistic about life, emotionality, nonetheless, represents being empathic, clear about people's feelings, and having the ability to communicate feelings to others. The self-control factor is characterized by the ability to regulate emotions and stress. Sociability, on the other hand, pertains to the ability to influence other people's feelings, defend your own rights, and maintain social awareness.

TEI has a very vital role in all domains of knowledge. In the domain of language learning, it has been found to be mostly related to language learner emotions. In this regard, higher levels of TEI were found to be linked to higher levels of learner positive emotions like enjoyment (Dewaele and Mercer, 2018; Li and Xu, 2019; Li, 2020; Resnik and Dewaele, 2020) and lower levels of negative emotions, such as anxiety (Dewaele et al., 2008; Dewaele, 2013; Shao et al., 2013; Li and Xu, 2019; Li, 2020) and boredom (Li et al., 2020). A positive relationship between TEI and enjoyment has been acknowledged by past research. In a 6-week EI intervention study among 56 high school Chinese students, Li and Xu (2019) found that intervention was effective in boosting learners' positive emotions (e.g., enjoyment), counteracting their negative ones (e.g., anxiety), and improving their EI. In addition, the results of the study by Li (2020) demonstrated that emotional intelligence is a positive predictor of enjoyment, and that enjoyment partially mediated the relationship between TEI and students' self-reported and actual language performance (Shao et al., 2020).

In addition to its positive links with PEs, research emphasized that TEI is significant in regulating emotions in that L2 learners with higher emotional intelligence perceived themselves as more capable of gauging the emotions of their interlocutor, controlling their stress, and feeling self-confident compared with those with lower emotional intelligence (e.g., Dewaele et al., 2008; Dewaele, 2013). In the same vein, Lake (2013) added that TEI helps language learners to recognize their own and others' strengths, overcome language obstacles, and obtain optimal affective and learning experiences in the L2 classroom.

TEI does not only help in regulating emotions but further enables individuals to transform negative emotions into positive emotions to reduce stress, anxiety, and conflict; improve relationships; and increase achievement, stability, self-motivation, social awareness, and harmony (see Goleman, 2012; Oxford, 2016). Furthermore, TEI has been established to be positively associated with learner resilience in learning (see Fiorilli et al., 2020; Trigueros et al., 2020).

### Language emotions

While past research (e.g., Gkonou et al., 2017) confirms the adverse effects of NEs (e.g., anxiety and boredom) on foreign language (FL) learning outcomes, the role of PEs (e.g., enjoyment) has only recently gained increasing momentum, which has been inspired by advances in the role of PP (Seligman and Csikszentmihalyi, 2000; Dewaele and MacIntyre, 2014, 2016; Gregersen and MacIntyre, 2014; Oxford, 2016; Gkonou et al., 2017). In this regard, research acknowledged that PEs enhance learners' resilience and persistence in facing the problems they encounter while learning a foreign language (Dewaele and MacIntyre, 2014; Dewaele et al., 2019). Additionally, MacIntyre and Gregersen (2012) recognized that PEs support broadening language learner cognition, controlling negative emotions, endorsing resilience, building personal and social resources, and generating greater wellbeing. One of the positive emotions mostly experienced by L2 learners is foreign language enjoyment. Seligman and Csikszentmihalyi, 2000 conceptualized enjoyment as “the good feelings people experience when they break through the limits of homeostasis–when they do something that stretches them beyond what they were” (p. 12). The positive effect of enjoyment in language learning goes beyond creating an enjoyable and safe psychological atmosphere for language learners to promoting their resilience and persistence in dealing with the difficulties they go through in FL learning. A vast line of research in the L2 domain acknowledged that EFL learners with higher levels of enjoyment appear more resilient in L2 learning, thereby acknowledging a positive connection between these two variables (Frederickson et al., 2003; MacIntyre and Gregersen, 2012; Dewaele and MacIntyre, 2014; Oxford, 2016; Dewaele and Alfawzan, 2018; Dewaele et al., 2018; MacIntyre et al., 2019; Shao et al., 2020). The influence of enjoyment in the course of L2 learning is usually further extended to alleviate the undesirable influence of NES with which enjoyment is often negatively connected. In this respect, learners who demonstrate high enjoyment usually display low levels of foreign language classroom anxiety as recognized by a vast line of L2 research (Dewaele and MacIntyre, 2014, 2016; Dewaele and Dewaele, 2017; Khajavy et al., 2018; Li et al., 2018; Jiang and Dewaele, 2019; Li and Xu, 2019; Elahi et al., 2021) and boredom (Dewaele et al., 2018; Dewaele and Li, 2022; Li and Wei, 2022). Furthermore, enjoyment has also been found to be linked to the effort learners expend in learning a foreign language (Pekrun and Linnenbrink-Garcia, 2014).

One of the most experienced NEs in the language classroom is anxiety. According to, Horwitz (1986), language anxiety is a specific state of anxiety learners experience when they participate in learning and/or using a language. Past studies have shown that anxiety is negatively related to different variables in the process of language learning (Gregersen and MacIntyre, 2014; Jiang and Li, 2017; Saito et al., 2018; Li et al., 2020). In such a process, anxiety has been found to be negatively correlated with positive emotions, such as enjoyment as established by a vast body of literature (e.g., Dewaele and Alfawzan, 2018; Jin and Zhang, 2018; Saito et al., 2018; Li et al., 2020). The research investigated the interplay between these two emotions and found a moderate negative correlation between anxiety and enjoyment. The findings of their study showed that it is possible to experience both a high level of enjoyment and anxiety or neither one nor the other (Resnik and Dewaele, 2020) concluding that enjoyment and anxiety are separate emotions instead of two opposite ends along the same continuum (Li et al., 2020) since high score on one variable does not automatically imply a low score on the other emotion.

Besides its negative associations with PEs, negative correlations have been established by earlier research between TEI and anxiety. For example, in a study involving 464 multilingual learners, Dewaele et al. (2008) found that those who scored higher on TEI reported significantly lower anxiety when using their different languages in a variety of situations attributing that to increased learner confidence in the ability to convey and read their emotions. Research shows that students' higher levels of trait emotional intelligence (TEI) were linked to lower levels of anxiety. In addition, TEI was revealed as a significant predictor of language anxiety (Resnik and Dewaele, 2020).

Similar to its negative connections with PEs and TEI, anxiety holds a negative relationship with L2 learner resilience. According to Chaffee et al. (2014) and Kim and Kim (2017), higher levels of language anxiety are often represented in low resilience in language learning. In this study, researchers maintained that the other side of the relationship is correct in that higher levels of resilience could play an important role in reducing learner anxiety associated with learning an L2. Although anxiety has been recognized as significantly adversely related to language achievement, researchers contested its negative effect over time (Alamer and Lee, 2021; Sparks and Alamer, 2022). For example, using cross-lagged panel analysis, Alamer and Lee (2021) found that a decrease in anxiety depends on increasing achievement and not the other around.

Foreign Language Classroom Boredom which was conceptualized by Pawlak et al. (2020) “as a negative emotion composed of disengagement, dissatisfaction, attention deficit, altered time perception and decreased vitality” (p. 2) is another NE that is highly associated with negative feelings, such as declined self-regulation, demotivation (Kruk and Zawodniak, 2018), low persistence, low activation and lack of interest, restlessness, anxiety, frustration, helplessness, dislike, unpleasant state of passiveness, guilt, tiredness, sleepiness, disengagement, and dissatisfaction (Li et al., 2021). Li et al. (2021), illuminated that such negative feelings and behaviors usually arise from class activities that are perceived by learners as over-challenging or under-challenging (repetitive, monotonous, undiversified) and/or unimportant, irrelevant, uninteresting, meaningless, and insufficiently stimulating. Past research has recognized the positive association between the negative emotions of anxiety and Boredom in that higher levels of anxiety are usually linked with higher boredom and vice versa (Kruk and Zawodniak, 2018; Li and Dewaele, 2020; Pawlak et al., 2020).

The negative emotions of anxiety and Boredom are often negatively linked with learner resilience whereas highly anxious and bored EFL learners exhibit lower resilience in L2 learning (e.g., Connor and Davidson, 2003; Chen and Padilla, 2019; Shao et al., 2020). In addition, Li and Dewaele (2020) examined the predictive effects of TEI and online learning achievement perceptions on the Boredom of 348 Chinese tertiary students. In this study, TEI and achievement perceptions co-predicted Boredom negatively confirming the negative association among these three constructs.

### Effort

Karabiyik and Mirici (2018) conceptualized effort in language learning as the amount of time and energy students invest to learn a foreign language and engaging students to fulfill the requirements of learning this language. According to Alamer (2021) and Hiver et al. (2021), language learners become more successful in learning a foreign language when they invest more effort and penitence in learning this language. In another study, Alamer (2022a) found that the nature and quality of the expended effort are much more important to recognize than the amount of effort being exerted. Investing effort alone could not, nonetheless, lead to successful language learning as established by earlier studies (Oxford and Shearin, 1994; Gardner, 2010; Alamer, 2022a,b) unless such effort is associated with strong positive psychological emotions for learning and engagement. With regard to learner emotions, the effort that learners invest in learning an L2 has been found to positively affect their enjoyment (Pekrun and Linnenbrink-Garcia, 2014; MacIntyre et al., 2019; Shao et al., 2020) and resilience (Kim and Kim, 2017). In this respect, we believe that effort is generally thought to be a basis for resilience that precedes it, and that the degree of resilience students demonstrate in an L2 is somehow dependent on the amount and quality of effort those students exert in learning this language. On the other hand, the effort is usually negatively related to learner negative emotions in that learners who devote much effort to language learning usually demonstrate lower levels of anxiety (Piniel and Csizér, 2013) and boredom (Pawlak et al., 2020) in the language classroom. However, these conclusions are only theoretically maintained, and it is necessary to examine the complex relationship between effort and language learner emotions and to assess how such a relationship might account for L2 resilience.

### L2 resilience

The psychological construct of resilience has been recently adopted in the EFL learning context (Kim et al., 2017). Kim and Kim (2019) emphasized that resilience augments people's ability to overcome difficulties and adversity and to interpret adversity positively through efforts rather than giving up. Among the characteristics that denote L2 learners' classroom resilience, according to Kim et al. (2017), are learner emotional positivity/ happiness (i.e., learners' perceptions of their lives as positive and satisfactory), persistence (paying continuous efforts to solve problems in the face of difficulties), and self-regulation (the ability to regulate one's own thoughts, feelings, and emotions). In EFL learning contexts, resilience has been found to have positive links with positive emotions like enjoyment, negative correlations with negative emotions like anxiety, and positive links with learner TEI as established earlier in this section.

### Hypothesized conceptual model

Theoretical links have been established by past research between enjoyment as a positive learner emotion and the character strengths of TEI (Dewaele and Mercer, 2018; Li and Xu, 2019; Li, 2020; Resnik and Dewaele, 2020), and effort (Pekrun and Linnenbrink-Garcia, 2014; MacIntyre et al., 2019; Shao et al., 2020). Accordingly, direct positive paths were depicted from TEI and effort to enjoyment leading to resilience. Besides, direct positive paths were also anticipated from TEI, effort, and enjoyment to resilience in light of the theoretical assumptions grounded in the literature that higher levels of TEI (Fiorilli et al., 2020; Trigueros et al., 2020), effort (Kim et al., 2017), and enjoyment (e.g., Frederickson et al., 2003; MacIntyre and Gregersen, 2012; Dewaele and MacIntyre, 2014; Oxford, 2016; Dewaele et al., 2018; MacIntyre et al., 2019; Shao et al., 2020) are usually associated with higher levels of resilience in L2 learning.

Because learners with a higher sense of character strengths like TEI and effort were generally found to experience a lower level of negative emotions, such as anxiety and Boredom (see e.g., Dewaele et al., 2008; Dewaele, 2013; Piniel and Csizér, 2013; Shao et al., 2013; Li and Xu, 2019; Li, 2020; Pawlak et al., 2020), negative direct paths from TEI and effort to anxiety and to boredom were drawn.

Since the feelings of language anxiety are usually equipped with a sense of boredom in L2 classes (Kruk and Zawodniak, 2018; Li and Dewaele, 2020; Pawlak et al., 2020), a direct positive path was drawn between these two variables. In addition, due to the well-acknowledged negative associations between negative and positive emotions, negative paths were predicted from anxiety to enjoyment (e.g., Dewaele and Alfawzan, 2018; Saito et al., 2018; Li et al., 2020; Moskowitz and Dewaele, 2020; among many others), as well as from boredom to enjoyment (Dewaele and Li, 2022; Li and Wei, 2022). Likewise, similar negative paths were estimated from anxiety and boredom to resilience based on theoretical argumentations made by past investigations (e.g., Connor and Davidson, 2003; Chen and Padilla, 2019; Shao et al., 2020).

## Method

### Participants

The present study involved 484 (308 female and 176 male) Saudi undergraduate students studying English at a public Saudi university. They were aged between 18 and 25 years, with a mean age of 20.2 years (*SD* = 0.48). Participants hold similar EFL learning experience (*M* = 8.85 years, *SD* = 0.49) and were studying at similar levels at the department and thus are believed to demonstrate similar levels of English proficiency.

Participants were invited *via* email to participate in this study by completing an online survey. Once consent to participate in the research was granted, a web link containing the online survey was provided to the study participants. Those who showed a willingness to withdraw from the study were advised to simply refrain from completing the questionnaire and leave the web page. An email was sent to all students in the English department, inviting them to participate in the study by completing an online survey *via* a link attached to that email. Those who started filling out the questionnaire and changed their mind about participation were asked to leave the web page.

### Measures

#### Foreign language enjoyment

Learner enjoyment in the FL classroom was assessed based on three factors: private/personal, Social, and Teacher support enjoyment. The 18 items used in this scale were adopted from previous studies on enjoyment (e.g., Dewaele and Alfawzan, 2018; Jin and Zhang, 2018; Li et al., 2018; Jiang and Dewaele, 2019).

#### Boredom

An 18-item scale was adapted by Li et al. (2021) to assess three factors pertaining to learner boredom in foreign language classes: foreign language classroom boredom, teacher-dislike boredom, and task boredom.

#### Anxiety

A total of 8 items that were extracted from the foreign language classroom anxiety scale by Horwitz (1986) were used in this study to capture the physical symptoms of learner anxiety, nervousness, and lack of confidence in the FL class. Two anxiety items were phrased to indicate low anxiety and were thus reverse coded so that high scores point to high anxiety for all items on this measurement.

#### Effort

Learners' effort was assessed using 10 items taken from Gardner's (2010) scale of effort and desire to learn English.

Students' enjoyment, boredom, anxiety, and effort were rated based on a seven-point agreement scale that ranges from totally agree (7) to totally disagree (1); with high scores indicating a high degree of enjoyment and effort and low degree of boredom and anxiety and vice versa. Negatively-worded items were assigned the opposite values in all scales.

#### Trait emotional intelligence

The Trait emotional intelligence questionnaire—-short form (TEIQue–SF) developed by Petrides (2009) and employed by Dewaele (2019) in the EF learning domain was used in this study. This 30-item scale comprises 15 facets (two items per facet) to assess five TEI factors: wellbeing, self-control, sociability, emotionality, and global TEI. Similar to the other scales, students were asked to display the degree of their agreement or disagreement with each statement in this measurement base on a 7-point Likert type scale ranging from “Completely Disagree” (1) to “Completely Agree” (7). Fifteen items of the scale were negatively worded and had to be reverse-coded and, consequently, high scores equal high TEI.

#### Resilience

A total of 15 items were originally adapted from Shin et al. (2009) and later validated by Kim et al. (2017) and were used in this study to obtain information about L2 learners' resilience. These items pertain to learner happiness (9 items), persistence (3 items), and self-regulation (3 items) and they were measured by a five-point Likert scale, ranging from *strongly disagree (1)* to *strongly agree (5)*.

The full survey is available in the online Supplementary material.

### Statistical analysis

The current research applied the partial least squares structural equation modeling (PLS-SEM) to assess the hypothesized model. PLS-SEM is an alternative approach to the widely used covariance-based SEM (CB-SEM) in that it focuses on explaining the variance in the outcome variables in the model (Hair et al., 2019). As such, PLS-SEM is most suitable when the research goal is explaining and predicting the outcome variables (exploratory) rather than theory testing (confirmatory) (Hair and Alamer, 2022). It is also a suitable SEM alternative when the model involves formative constructs, i.e., constructs that are defined by their indicators and not the other way around (see Sparks and Alamer, 2022 for a detailed example). In contrast to reflective constructs, formative constructs are evaluated by two major steps: (1) the indicators multicollinearity with the variance inflation factor (VIF) with values below 5 indicating no issue of multicollinearity in the model, and (2) the size and significance of the indicator weights and loadings (Hair and Alamer, 2022).

Because PLS-SEM is a variance-based method, the evaluation of the model relies on several prediction measures, not on the goodness of fit (Hair et al., 2019). However, we report the most commonly used model fit index within the PLS domain, the standardized root mean square residual (SRMR), and its exact fit, with values below 0.08 indicating acceptable fit (Hair and Alamer, 2022). Two major criteria were used: the explanatory power, which is assessed *via* the coefficient of determination (the *R*^2^ value), and the predictive power, which is assessed through the PLS_predict_ procedure (Shmueli et al., 2019). The *R*^2^ value is interprated using Hair and Alamer (2022) as follows: values of 0.06, 0.16, and 0.36 are indicative of small, medium, and strong explanatory power, respectively. To provide information about model out-of-sample predictive capability, PLS_predict_ procedures “incorporate model assessments based on an initial training sample (randomly drawn separate sub-sample of the total sample) and estimate the predictive power of the model on a second hold-out sample of data–other than that used in calculating the initial PLS-SEM solution” (Hair and Alamer, 2022, p. 8). In this way, PLS structural model has a predictive power if the errors (i.e., RMSE) it produces are less than the errors produced by the naïve linear regression model (LM) (for greater details see Shmueli et al., 2019). An assessment of collinearity was also considered using the VIF index. Also, following Hair and Alamer's (2022) guidelines in interpreting the effect size of the structural model, β values in the ranges of 0–0.1, 0.1–0.3, and 0.3–0.5, and those that are >0.5 are indicative of weak, modest, moderate, and strong effect sizes, respectively.

## Results

### Missing values, outliers, and normality

The data of the present study did not contain any missing values. Another check through Q-Q plots has been done to check if there were any outlier values in the data that depart significantly from the rest. The results did not show any problematic values. Normality was assessed by inspecting skewness and kurtosis values following the +2/-2 guidelines (Hair et al., 2022). The data did not show a departure from these cut-off values.

### Assessing the measurement model

As shown in Table 1, the data appears to be relatively normally distributed. In the structural model, the constructs were measured formatively. That is, specific constructs (e.g., private/personal enjoyment, social enjoyment, and Teacher support enjoyment) are specified to affect the general construct (e.g., enjoyment). Because these sub-factors are not interchangeable it was believed that formative formulation is a more appropriate model specification. Following Hair and Alamer (2022) recommendations, we evaluated the validity of the model through collinearity and the weights of formative indicators. All VIF values were below the cut-off value of 5 and only two indicators, “emotional regulation” and “emotional positivity” exceeded 3 but did not reach 5. We concluded that there is no critical issue of multicollinearity in the model and that the indicators uniquely measure their constructs. With respect to significance testing, we found that all outer weights have shown positive and significant weights with the exception of three constricts “Task boredom,” “Persistence” and “Self-control” as their outer weights were found to be non-significant. We inspected their outer loadings and found that their loadings were >0.60 with *p* < 0.001. We retain these indicators and conclude that they are important but not substantially relevant. Note that the assessment of redundancy analysis (i.e., convergent validity) for the formative constructs was not possible due to the lack of a global single-item measure in our data.

**Table 1:** Descriptive statistics, normality, and correlation matrix.

	**TEI**	**Resilience**	**Effort**	**Enjoyment**	**Anxiety**	**Boredom**
TEI	4.66					
Resilience	0.28***	3.54				
Effort	0.45***	0.39***	3.76			
Enjoyment	0.39***	0.59***	0.45***	3.74		
Anxiety	−0.25***	−0.25***	−0.24***	−0.31***	3.05	
Boredom	−0.36***	−0.52***	−0.39***	−0.58***	0.53***	2.88
Skew/Kurt	0.35/−0.54	−0.43/−0.69	−0.66/1.22	−0.29/−0.75	−0.01/−0.65	−0.31/−0.56

### Assessing the structural model

**Figure 1:**
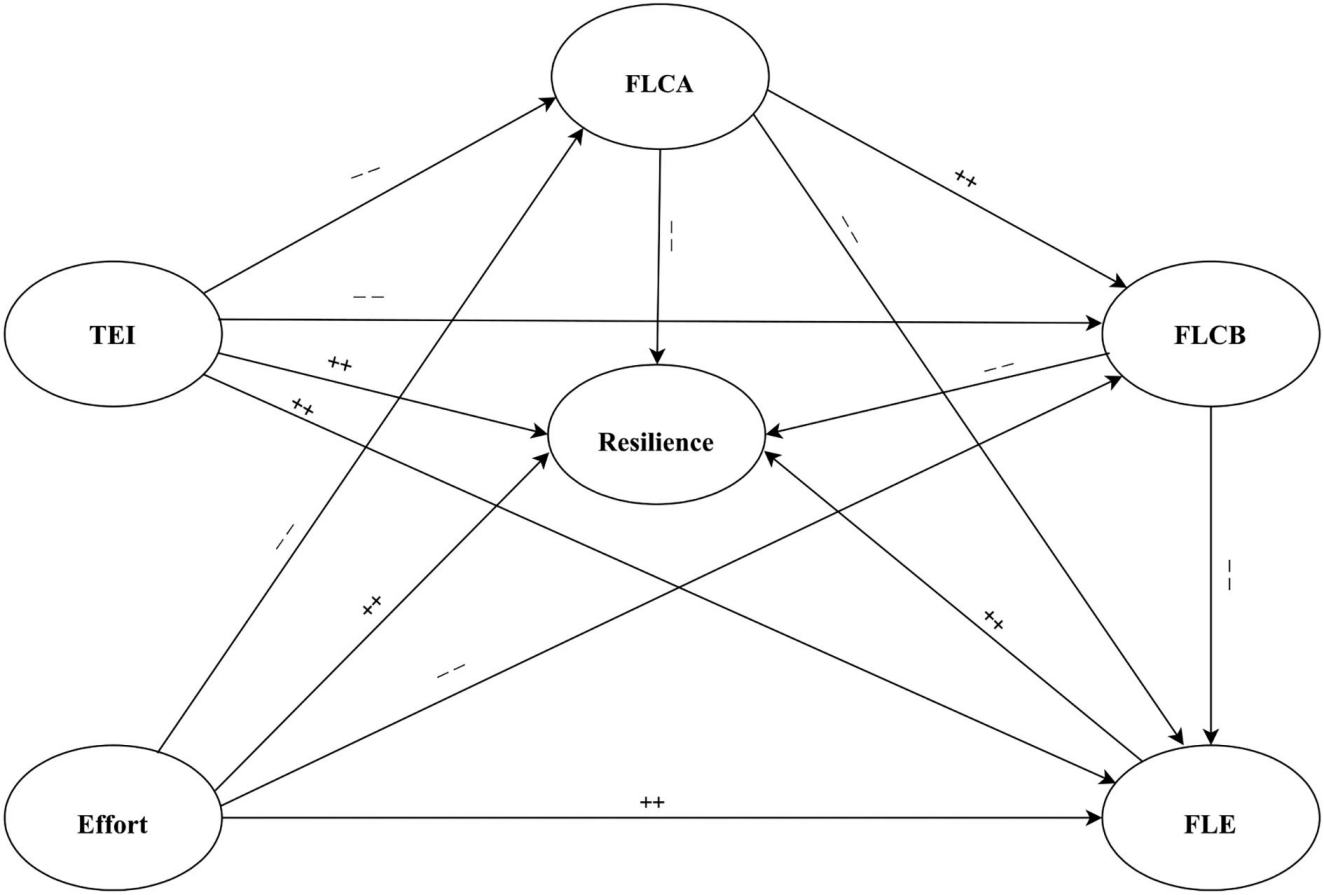
The structural (conceptual) model links the variables to resilience. ++ = a positive path, −− = a negative path.

**Figure 2:**
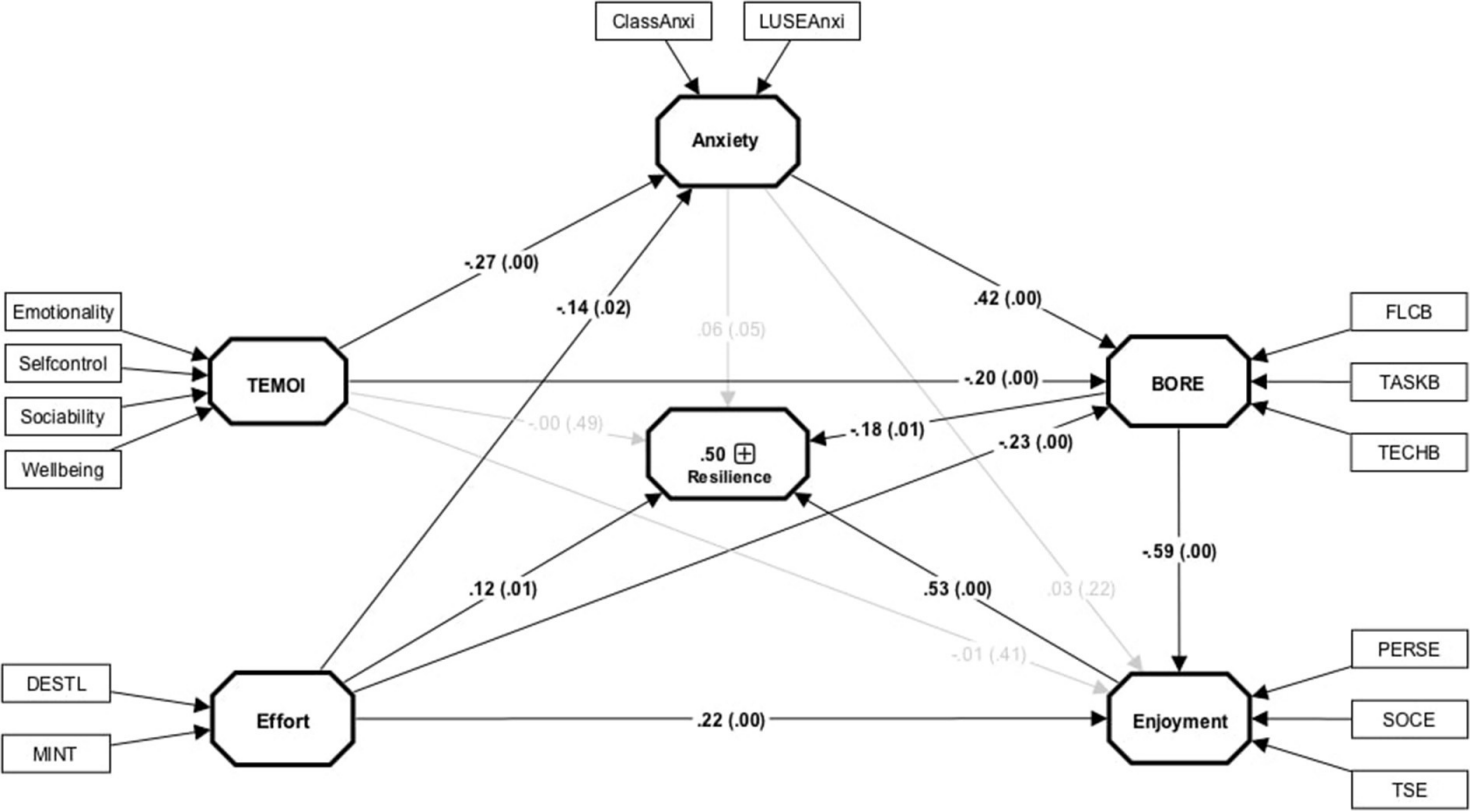
The results of the structural model. Non-significant paths are indicated using gray lines; values in brackets are bias corrected confidence interval (CI) 95%. TEMOI, Trait Emotional Intelligence; BORE, Boredom; ClassAnxi, Classroom Anxiety; LUSEAnxi, Language Use Anxiety; FLCB, Foriegn Language Classroom Boredom; TASKB, Task-related Boredom; TECHB, Teacher-related Boredom; BERSE, Personal Enjoyment; SOCE, Social Enjoyment; TSE, Teacher-related Enjoyment; DESTL, Desire to learn the Language; MINT, Motivational Intensity.

The assessment of the structural model presented in Figure 1 starts by reporting the SRMR fit index. The results show that the SRMR of our structural model was 0.07 (HI 95% CI = 0.03), suggesting an approximate fit to the data. We also evaluate the collinearity by inspecting the VIF values. The results showed that all values were below 3, with a maximum value was 2.40 which is way below the cut-off value of 5. We conclude that empirical evidence suggests that the constructs are distinct, thus discriminant validity is established. Next, we assessed the explanatory power of the model with a special focus on the outcome variable, “resilience.” The results of the structural model can be seen in Figure 2. In this way, the analysis showed that the model explained around 50% [95% CI: 40, 0.55] of the variance in the outcome. According to Hair and Alamer (2022), this value indicates a strong explanatory power of the model for the outcome. The results of the PLS_predict_ have been, then considered. Because the outcome variable consists of three indicators, the PLS_predict_ assesses the RMSE values on these indicators. Table 2 presents the results of the RMSE in the two models and shows that all indicators in the PLS model have generated the almost exactly same amount of prediction error. Following Hair and Alamer (2022), the results of PLS_predict_ suggest that the structural model has a moderate out-of-sample predictive power.

**Table 2:** The results of PLS_predict_ analysis.

**Indicators of the outcome (resilience)**	**RMSE value**	
	**PLS model**	**LM model**
Happiness	0.76	0.76
Persistence	0.85	0.85
Self-regulation	0.93	0.93

After assessing the overall model, we evaluated the path coefficients for their size and significance. Direct effects between the variables are illustrated in Figure 2 while indirect and total effects are presented in Table 3. Several pathways were found meaningful in the model, and we highlight these accordingly (see Table 3 for full details of mediation). The model indicates that TEI did not relate directly to resilience. Its effect, however, was fully mediated through learner emotions of enjoyment, anxiety, and boredom (see Table 3 for full details of mediation). Moreover, the total effect of TEI on resilience was significant but modest in size (β = 0.13, *p* = 0.003). Similarly, anxiety was not related directly to resilience or enjoyment. Its effect on the outcome ‘resilience' was only indirectly detected *via* boredom and enjoyment. Anxiety total effect was positive but modest in magnitude (β = −0.13, *p* = 0.004). Furthermore, ‘effort' appears to be associated with resilience directly and indirectly through boredom and enjoyment. The total effect of ‘effort' was moderate in size (β = 0.36, *p* < 0.001). Moreover, the effect of boredom on the outcome ‘resilience' was direct and indirect through enjoyment. Its total effect was strong (β = 0.50, *p* < 0.001). In addition, enjoyment has a positive and strong effect on resilience (β = 0.53, *p* < 0.001), and no indirect effect was postulated with this variable.

**Table 3:** Indirect and total effects of the variables on the outcome.

**Indirect paths**	**β**	***p***
		
TEI -> Boredom -> Resilience	0.04	0.03
TEI -> Boredom -> Enjoyment -> Resilience	0.06	0.001
TEI -> Anxiety -> Boredom -> Resilience	0.02	0.04
TEI -> Anxiety -> Boredom -> Enjoyment -> Resilience	0.04	0.001
Effort -> Boredom -> Resilience	0.04	0.02
Effort -> Enjoyment -> Resilience	0.12	< 0.001
Effort -> Boredom -> Enjoyment -> Resilience	0.07	< 0.001
Boredom -> Enjoyment -> Resilience	−0.31	< 0.001
Anxiety -> Boredom -> Resilience	−0.07	0.02
Anxiety -> Boredom -> Enjoyment -> Resilience	−0.13	< 0.001
Total effects
Anxiety	−0.13	0.004
Boredom	−0.50	< 0.001
Enjoyment	0.53	< 0.001
Effort	0.36	< 0.001
TEI	0.13	0.003

The 95% CI are based on bias corrected method.

## Discussion

Previous research has established that TEI of language learners is associated with positive (Chow et al., 2018; Dewaele and Alfawzan, 2018; Saito et al., 2018) and negative emotions (Dewaele et al., 2019; Pawlak et al., 2020; Li et al., 2021) as well as their resilience in language learning (Fiorilli et al., 2020; Trigueros et al., 2020). Despite that, only a few studies have illustrated the mediational process of how character strengths might facilitate learners' L2 emotions and eventually their L2 resilience. The primary goal of the present research was to identify and elaborate on the interrelationship among L2 learner character strengths of TEI and effort, classroom emotions, and resilience in language learning *via* a comprehensive model from a positive psychology perspective. To fully understand the complex relationship between the constructs, we applied PLS-SEM to study the structural relationships and assess their external validity through predictive assessment (Alamer, 2022b; Hair and Alamer, 2022). Our selection of PLS-SEM was also justified given the nature of the constructs involved in the assessment as they were operationalized as formative constructs. Specifically, using higher-order factor models where the lower-order factors are affecting (or causing) the higher-order factor are not directly possible in the way we tested the mode in CB-SEM. Thus, PLS-SEM was an approparite selectnion for such a case (Hair et al., 2019).

The findings of this study revealed that our model shows adequate applicability and fit the data and accounted for 50% of learner L2 resilience. Thus, the hypothesized model can be better generalized to language learning contexts. By testing this structural model, it is clear that L2 learner TEI had no significant direct associations with learner positive emotion of enjoyment as well as with the outcome variable in the model (i.e., L2 resilience). These findings run counter to theoretical conclusions made by past research emphasizing that, in the course of language learning, learners' TEI is usually associated with their classroom enjoyment (e.g., Li and Xu, 2019; Li, 2020; Shao et al., 2020) and L2 resilience. It is noteworthy mentioning, nevertheless, that a direct negative relationship between TEI to negative emotions was depicted in this study. Our results, therefore, provide support for the direct negative associations identified by past research (e.g., Dewaele et al., 2008; Dewaele, 2013; Piniel and Csizér, 2013; Shao et al., 2013; Li and Xu, 2019; Li, 2020; Pawlak et al., 2020) between learner character strengths of TEI and negative emotions of anxiety and boredom confirming that learners with higher levels of TEI usually demonstrate lower levels of negative emotions, such as anxiety and boredom, and vice versa. While past research has well-acknowledged the bidimensional role of TEI in both regulating NEs (e.g., Dewaele et al., 2008; Dewaele, 2013; Lake, 2013) and boosting PEs (Dewaele and Mercer, 2018; Li and Xu, 2019; Li, 2020; Resnik and Dewaele, 2020), the data of our model showed that learner TEI adopt the former rather than the latter role (i.e., regulating learner NEs rather than promoting PEs). This could emphasize the importance of self-control as a significant factor of TEI which considers emotion regulation and alleviating the negative effects of negative emotions a prerequisite step for enhancing the positive side of learner emotions. This has been established by (e.g., Dewaele et al., 2008; Dewaele, 2013; Li and Xu, 2019) who illuminated that TEI is largely about regulating emotions and that developing greater TEI helps learners in primarily regulating their NEs, and in turn, transforming their NEs into PEs, and accordingly improving learning outcomes like achievement and resilience (Oxford, 2016; Li and Xu, 2019).

While no direct relationship between TEI and the outcome variable (i.e., resilience) was established in our model, such a relationship was captured through the mediation of boredom as a negative emotion, the combined role of negative emotions of boredom and anxiety, and the combined role of a mix of negative and positive emotions (e.g., boredom and enjoyment, anxiety, boredom, and enjoyment, etc.). This emphasizes that learner emotions can hold multiple functions in determining the relationship between learner strengths and learner L2 resilience in that they are not only directly associated with their TEI but also facilitate the relationship between learner TEI and their resilience. Such findings cooperate with those of earlier investigations where negative emotions, namely anxiety, mediated the relationship between EI and outcome variables (e.g., L2 achievement) as in the studies of Shao et al. (2013) and Li (2020). In addition, the coexistence of both negative and positive emotions as co-mediators of the relationship between TEI and L2 resilience in the present study is similar to the mediation model in the study of Li (2020) where negative and positive emotions co-mediated the relationship between TEI and L2 achievement confirming that learner negative and positive emotions could interchangeably interact not only to predict L2 learning outcomes but also to account for the relationship between L2 learner character strengths and such outcomes.

Effort, the second character strength, appeared to play a greater role in predicting L2 resilience than TEI. In this respect, the effort had a positive direct association with L2 resilience. This supports the very few theoretical conclusions available (Kim et al., 2017) about the strong connections between effort and resilience in language learning in the way that learners devoting a larger amount of effort to learning a foreign language usually tend to exhibit higher levels of resilience in learning that language. Besides its direct negative links with L2 resilience, the effort had a significant positive relationship with enjoyment as a positive emotion indicating that learners who expend greater effort in language learning display higher levels of enjoyment in learning that language as theoretically hypothesized by earlier L2 investigations (see Pekrun and Linnenbrink-Garcia, 2014; MacIntyre et al., 2019; Shao et al., 2020). In addition, negative connections have been detected between effort and the negative emotions of anxiety and boredom in predicting learner resilience. Such negative connections support the theoretical claims in the literature that learners who devote much effort to learning an L2 usually demonstrate lower levels of anxiety (Piniel and Csizér, 2013) and boredom (Pawlak et al., 2020) while learning this language. Moreover, the effort did not only associate with negative and positive emotions in predicting L2 resilience but also significantly indirectly accounted for learner resilience *via* the mediation of learner enjoyment as a positive emotion, boredom as a negative emotion, and the mixed positive and negative effect of these two emotions. This yet again emphasizes the vital mediating role of learner emotions in accounting for the relationship between learner character strengths and L2 learning outcomes. It establishes that, in the course of language learning, learners devoting much effort to L2 learning are expected to show a higher degree of L2 resilience, dependent on those learners experiencing higher EFL and lower boredom in their EFL classes.

As anticipated in the hypothesized model, the negative emotion of boredom had a negative direct negative influence on L2 resilience revealing that the degree of resilience L2 learners demonstrate in L2 learning is adversely influenced by the sense of boredom they experience in language class (e.g., Shao et al., 2020). In addition, boredom had also a strong direct negative effect on learner enjoyment recognizing the conclusions made by earlier investigations that the learners' feelings of boredom in language class usually undermine their sense of enjoyment in learning an L2.

Despite that the other negative emotion (i.e., anxiety) had a positive direct influence on boredom confirming what has been hypothesized by past theoretical and empirical research that the feelings of language anxiety are usually coupled with a sense of boredom (Kruk and Zawodniak, 2018; Li and Dewaele, 2020; Pawlak et al., 2020), anxiety did not reach a significant level in directly predicting learner L2 resilience. In this study, the role of anxiety in predicting L2 resilience has not been unique but rather dependent on other learner emotions where it has only indirectly affected L2 resilience through the sole effect of boredom and the combined influence of boredom and enjoyment. Thus, it can be argued that anxiety in this study context might not be necessarily harmful to learner resilience but rather the increasing feelings of boredom and lack of enjoyment, likely resulting from anxiety, are responsible for affecting L2 resilience in this model. The absence of a direct relationship between learner anxiety and L2 resilience in our model establishing that learner feelings of language anxiety have no direct role to play in their resilience in language learning appears an uncommon result given the vital role of anxiety in affecting different aspects of language learning (see Gregersen and MacIntyre, 2014; Jiang and Li, 2017; Dewaele et al., 2018; Saito et al., 2018; Li et al., 2020) including learner resilience (e.g., Chaffee et al., 2014). It, however, goes in line with what recent investigations such as that of Sparks and Alamer (2022) as well as Alamer and Lee (2021) found with L2 students as they reported that anxiety does not necessarily impact language learning, but rather might be a consequence of poor learning. This conclusion, however, merits further validation by future investigations in other EFL/ESL contexts.

Interestingly, the model in this study showed that L2 resilience has been directly positively influenced by learner enjoyment postulating that the more L2 learners feel enjoying L2 learning, the higher resilient they appear to be in learning this language (e.g., Frederickson et al., 2003; MacIntyre and Gregersen, 2012; Dewaele and MacIntyre, 2014; Oxford, 2016; Dewaele et al., 2018; MacIntyre et al., 2019; Shao et al., 2020). In addition to its direct influence on L2 resilience, enjoyment appeared to control the relationship between learner boredom and resilience establishing that the way that EFL learners' boredom affects their L2 resilience is determined by the degree of enjoyment those learners display. Another notable finding in the present study is that leaner enjoyment as a positive emotion had the largest total effect on learner resilience as well as the strongest direct influence on this variable. This conclusion once again indicates that learners' sense of enjoyment in language classes plays a major role in accounting for their resilience to learn a foreign language. This matches the findings of a vast body of research that acknowledge the crucial role of enjoyment in language learning in general (see Dewaele and Alfawzan, 2018; Dewaele et al., 2018; Li et al., 2019; MacIntyre et al., 2019; Shao et al., 2020) and in enhancing L2 learner resilience in particular. Importantly, the total predictive power of learner enjoyment of positive emotion was larger than that of boredom as a negative emotion in our model. This emphasizes that learners' L2 positive emotions play a better role in explaining their L2 resilience (see, Gkonou et al., 2017; Kim et al., 2017; Dewaele et al., 2018; Kim and Kim, 2019) than their negative emotions. It also verifies the claims that the role of positive emotions in language learning usually outweighs that of negative emotions (see MacIntyre and Gregersen, 2012; Dewaele et al., 2018, 2019; Alamer and Lee, 2019).

Overall, the findings that emerged out of the original model in this study substantially align with the positive psychological perspectives that recognize the vital role of character strengths and learner emotions in language learning in that such strengths and emotions help enhance L2 learner resilience. Most interestingly, while the total effect of TEI on L2 resilience was significant, the paths in the structural model showed that L2 learner TEI did not significantly explain their resilience directly but rather completely indirectly through the mediation of learner negative and positive emotions. TEI only directly correlated with the negative emotions of anxiety and boredom and influence L2 resilience through these two emotions besides enjoyment. This suggests that regulating learner emotions could be a precondition for learner TEI to have a role in their L2 resilience. Effort, the other character strength, showed a stronger role in predicting L2 resilience than TEI in that it, directly and indirectly, predicted learner resilience and, consequently, had a larger total effect on this variable than TEI; and also significantly directly correlated with learner both positive and negative emotions. Learner emotions of enjoyment and boredom scored the largest total effect on learner resilience in the model because they not only directly predicted L2 resilience, but also mediated the relationship between learner TEI and effort character strengths, the negative emotion of anxiety, and L2 resilience. This multidimensional role of enjoyment and boredom recognizes their crucial role in explaining learner resilience in language learning which deserves to attract the attention of future research in the L2 domain.

### Implications

It is undeniable that learner resilience plays an important role in the foreign language learning process. However, for this concept to properly operate in such a process, it should be accompanied by effective character strengths and positive emotions. This study aimed to test the complex relationship among a variety of variables representing language learner character strengths and emotions and to unveil the role of these variables in learner resilience for foreign language learning. The primary conclusion this study came up with acknowledged the positive association between learner character strengths and learner emotions and emphasized the bi-dimensional very significant role of these variables in language learning in that they help in enhancing L2 learner resilience.

The findings of this study suggest a number of pedagogical implications. Because learners who are emotionally engaged in language learning are usually less likely to experience negative emotions, L2 educators need to build on learners' character strengths and positive emotions to ease the negative consequences of negative emotions and in turn enhance their resilience in language learning. In this respect, L2 teachers can also get the benefit of deploying EI intervention programs such as the one utilized by Li and Xu (2019) with their EFL learners to improve their emotional experiences of those learners. Furthermore, deploying positive emotions interventions to promote PEs (with greater emphasis on enhancing learner enjoyment) might be very useful in controlling negative emotions and endorsing resilience and persistence in language learning as established by earlier research (MacIntyre and Gregersen, 2012; Dewaele et al., 2018, 2019; Kim and Kim, 2019). Besides promoting learners' positive emotions, L2 teachers should take whatever is necessary to regulate negative emotions, such as boredom and anxiety. One useful example in this regard is what MacIntyre (2016) recommended that teachers and learners can complement anxiety-reduction strategies with applications of character strengths, such as courage to undo the negative effects of language anxiety.

## Conclusion and limitations

The present study aims to model the relationships between learner character strengths (including TEI and effort) and classroom emotions (enjoyment, anxiety, and boredom) in language resilience. We applied SEM to test the hypothesized model and the results provided us with a greater understanding of how these variables are serially connected and lead to resilience. Nonetheless, a number of limitations must be presented. First, the present study applied the hypothesized model to one socio-educational context (i.e., Saudi learners of English). Thus, although the results can be generalized to learners with similar characteristics, they cannot be assumed to be the same across distinct socio-cultural contexts. It would be plausible to replicate the findings of the present study with other types of L2 learners to see the similarities and differences. Moreover, the present article employed only a cross-sectional survey design. Thus, any claims about cause-and-effect relationships among the predictors and outcomes were not assumed. The structural model was limited in that it depicts only how the variables are linked to each other. We suggest further research to assess the confirmed effects from a longitudinal perspective to reach a better, yet not full, understanding of causation. Lastly, the present study was based on self-report data, gathered from an online questionnaire. The respondents found the length of the survey a bit too long (about 100 items altogether), which might lead to inaccurate responses which is an inevitable challenge to the “truthfulness” in the responses. Overall, it is our hope that the information presented in this research can be disseminated to language education and positive psychology communities and be known to teachers and educators to be used to further the learning and teaching practices.

## Data availability statement

The original contributions presented in the study are included in the article/Supplementary material, further inquiries can be directed to the corresponding author/s.

## Ethics statement

Ethical review and approval was not required for the study on human participants in accordance with the local legislation and institutional requirements. The patients/participants provided their written informed consent to participate in this study.

## Author contributions

Both authors contributed to the article and approved the submitted version.

## Conflict of interest

The authors declare that the research was conducted in the absence of any commercial or financial relationships that could be construed as a potential conflict of interest.

## Publisher's note

All claims expressed in this article are solely those of the authors and do not necessarily represent those of their affiliated organizations, or those of the publisher, the editors and the reviewers. Any product that may be evaluated in this article, or claim that may be made by its manufacturer, is not guaranteed or endorsed by the publisher.
