# Supplementary material for: The role of learner character strengths and classroom emotions in L2 resilience
Source: Front Psychol. 2022 Oct 14;13:956216. doi: 10.3389/fpsyg.2022.956216 (PMC9614248; doi:10.3389/fpsyg.2022.956216)
Supplement: Supplementary file 1 [file Table_1.DOCX]

Supplementary Material

**Measures**

**Enjoyment**

1. I don’t get bored while learning English.

2. I enjoy learning English.

3. I feel as though I’m a different person during English language class.

4. I learnt to express myself better in English language.

5. I’m a worthy member of the English language class.

6. I’ve learnt interesting things in English language.

7. In English language class, I feel proud of my accomplishments.

8. Learning English is fun.

9. It is cool to know English as a foreign language.

10. The peers in my English language class are nice.

11. We form a tight group.

12. We laugh a lot in English class.

13. English class is a positive environment.

14. English class is fun.

15. There is a good atmosphere in English class.

16. My English language teacher is encouraging.

17. My English language teacher is friendly.

18. My English language teacher is supportive.

**Boredom**

1. The English class bores me.

2. I start yawning in English class because I’m so bored.

3. My mind begins to wander in the English class.

4. I am only physically in the classroom, while my mind is wandering outside the English class.

5. It is difficult for me to concentrate in the English class.

6. Time is dragging on in English class.

7. I get restless and can’t wait for the English class to end.

8. I always think about what else I might be doing to kill the time rather than sitting in

this English class.

9. I am not interested in English class, because the English teacher isn’t likable

(e. g., tone, pitch or facial appearance).

10. The English teacher is an uninteresting, so the English class is dull.

11. I really dislike the English teacher spending so much time making personal comments.

12. I feel agitated because the English teacher spends too much time saying things that

are irrelevant to the teaching material.

13. So many similar types of (English) exercises make me lose interest.

14. So much practice on a same (English-related) subject matter makes me restless.

15. The (English-related) exercise or a subject matter lasts too long, and I feel bored.

16. I don’t care about teaching and learning activities that the English teacher does not

value.

17. When the English teacher seems unmotivated to teach, I lose my motivation to

listen to him/her as well.

18. If I cannot understand classmates’ presentations, I become really bored.

**Anxiety**

1. Even if I am well prepared for FL class, I feel anxious about it.

2. I always feel that the other students speak the FL better than I do.

3. I can feel my heart pounding when I’m going to be called on in FL class.

4. I don’t worry about making mistakes in FL class (reverse-coded).

5 I feel confident when I speak in FL class (reverse-coded).

6. I get nervous and confused when I am speaking in my FL class.

7. I start to panic when I have to speak without preparation in FL class.

8. It embarrasses me to volunteer answers in my FL class.

**Effort**

1. I work really hard to learn English.

2. When I have a problem understanding something in my English class, I always ask my teacher for help.

3. When I’m studying English, I ignore distractions and pay attention to my task.

4. I make a point of trying to understand all the English I see and hear.

5. I keep up to date with English by working on it almost every day.

6. I don’t pay much attention to the feedback I receive in my English class.

7. I don’t bother checking my assignments when I get them back from my English teacher.

8. I put off my English homework as much as possible.

9. I tend to give up and not pay much attention when I don’t understand my English teacher’s explanation of something.

10. I can’t be bothered trying to understand the most complex aspects of English.

**Trait Emotional Intelligence**

1. Expressing my emotions with words is not a problem for me.

2. I often find it difficult to see things from another person’s viewpoint.

3. On the whole, I’m a highly motivated person.

4. I usually find it difficult to regulate my emotions.

5. I generally don’t find life enjoyable.

6. I can deal effectively with people.

7. I tend to change my mind frequently.

8. Many times, I can’t figure out what emotion I'm feeling.

9. I feel that I have a number of good qualities.

10. I often find it difficult to stand up for my rights.

11. I’m usually able to influence the way other people feel.

12. On the whole, I have a gloomy perspective on most things.

13. Those close to me often complain that I don’t treat them right.

14. I often find it difficult to adjust my life according to the circumstances.

15. On the whole, I’m able to deal with stress.

16. I often find it difficult to show my affection to those close to me.

17. I’m normally able to “get into someone’s shoes” and experience their emotions.

18. I normally find it difficult to keep myself motivated.

19. I’m usually able to find ways to control my emotions when I want to.

20. On the whole, I’m pleased with my life.

21. I would describe myself as a good negotiator.

22. I tend to get involved in things I later wish I could get out of.

23. I often pause and think about my feelings.

24. I believe I’m full of personal strengths.

25. I tend to “back down” even if I know I’m right.

26. I don’t seem to have any power at all over other people’s feelings.

27. I generally believe that things will work out fine in my life.

28. I find it difficult to bond well even with those close to me.

29. Generally, I’m able to adapt to new environments.

30. Others admire me for being relaxed.

**Resilience**

1. When I have a problem, I try to solve it after reflecting on the cause of the problem.

2. I first contemplate diverse possible solutions to a problem in order to solve it.

3. I can break through any distractions when having important things to do immediately.

4. I easily give up when things go wrong. (reverse-coded)

5. I would work harder.

6. I would keep trying

7. I would not change my long-term goals and ambitions.

8. I would look forward to showing that I can improve my grades.

9. I would do my best to stop thinking negative thoughts.

10. I believe that I am able to control my emotions when having difficulties.

11. I can regulate my feelings when having discussions with my classmates and teacher.

12. The conditions of my life are satisfactory.

13. If I write down all of the things that I feel grateful for, there will be a long list.

14. I am sure that everything will be fine even in difficult situations.

15. I think my hard work always pays off.
